# Socio-economic drivers of specialist anglers targeting the non-native European catfish (*Silurus glanis*) in the UK

**DOI:** 10.1371/journal.pone.0178805

**Published:** 2017-06-12

**Authors:** E. M. Ann Rees, V. Ronni Edmonds-Brown, M. Fasihul Alam, Ros M. Wright, J. Robert Britton, Gareth D. Davies, Ian G. Cowx

**Affiliations:** 1 Department of Life Sciences, Hertfordshire University, Hatfield, Hertfordshire, United Kingdom; 2 College of Human and Health Sciences, Swansea University, Singleton, Swansea, United Kingdom; 3 Fisheries Technical Services, Environment Agency, Kelvedon, Essex, United Kingdom; 4 Department of Life & Environmental Sciences, Bournemouth University, Poole, Dorset, United Kingdom; 5 National Fisheries Services, Environment Agency, Brampton, Cambridgeshire, United Kingdom; 6 School of Biological, Biomedical & Environmental Sciences, Hull International Fisheries Institute, Hull University, United Kingdom; Pacific Northwest National Laboratory, UNITED STATES

## Abstract

Information about the socioeconomic drivers of *Silurus glanis* anglers in the UK were collected using questionnaires from a cross section of mixed cyprinid fisheries to elucidate human dimensions in angling and non-native fisheries management. Respondents were predominantly male (95%), 30–40 years of age with <10 yr angling experience for *S*. *glanis*; most had received college rather than university education. The majority (34%) were employed with low-moderate income status (<£30k per annum), which may restrict time and expenditure spent on angling. Highest angling expenditure was on equipment and bait with most from southern England (54%) spending >£500 per annum. The proportion of time spent angling for *S*. *glanis* was significantly related to angler motivations; fish size, challenge in catch, tranquil natural surroundings, escape from daily stress and to be alone were considered important drivers of increased time spent angling. Overall, poor awareness of: the risks and adverse ecological impacts associated with introduced *S*. *glanis*, non-native fisheries legislation, problems in use of unlimited ground bait and high fish stocking rates in angling lakes were evident, possibly related to inadequate training and information provided by angling organisations to anglers, as many stated that they were insufficiently informed.

## Introduction

Globally, recreational angling represents a pastime with a high number of active participants, particularly in developed countries across northwest Europe and North America [1]. In Europe, there are around 25 million anglers whose activities play an important role in supporting regional and local economies, especially in rural areas [2]. For example, in England and Wales [3, 4] estimated total annual effort by licensed anglers was over 30 million angler days, with the associated expenditure relating to this activity being £1.18 billion.

Understanding the motivations and drivers of anglers contributing to this effort is important to ensure fishery managers implement measures to maintain or enhance angler satisfaction. It can also assist in the general management of exploited fish stocks [5] and help implement policies that minimise detrimental ecological impacts from fishery management activities [6]. This latter point is important given the increasing popularity of ‘big game’ type freshwater angling in lake fisheries in countries such as England and Wales [6]. In these fisheries, large-bodied non-native species tend to be introduced with the sole purpose of providing new and challenging angling experiences that are often completed without adequate ecological risk assessment [7]. Indeed, many of these introductions have been illegal and whose potentially irreversible ecological consequences have yet to be fully realised [6].

An example of a large-bodied, predatory, trophy fish increasingly used to enhance the performance of lake fisheries in England and Wales is the non-native European catfish *Silurus glanis* [8]. Its popularity stems from its large size relative to native fishes, with individuals now often released at weights above 27 kg (usually imported from mainland Europe), allied to the challenge of adept angling skills for capture, with anglers prepared to pay more in day tickets, and membership rates for access to *S*. *glanis* specimen lakes that can account for increased revenues in these fisheries [9, 10]. Present in England since the 19^th^ Century [11], the number of lake fisheries where *S*. *glanis* is present has increased to over 500 in the last 20 years with high fish stocking rates ~1000 kg ha^-1^in some syndicate waters [8, 12], which are mainly established in eastern England and in counties surrounding London, i.e. Hertfordshire, Bedfordshire, Essex, Kent and Sussex, although fisheries are increasingly found in the Midlands and northern England [8, 9].

Many *S*. *glanis* introductions are completed illegally, and some of the lake fisheries are in locations that enable natural dispersal, such as on floodplains, resulting in some individual fish dispersing into rivers during flood events [8, 13, 14]. In contrast to populations in Southern Europe, *S*. *glanis* has yet to establish invasive populations in England and Wales [8] because thermal constraints appear suboptimal for establishment [13, 15] but is present in several southern and eastern English rivers (Thames, Colne, Chelmer, Kennet, Ouse). This, however, is liable to change if individuals continue to disperse into rivers and summer water temperatures increase through climate change [14] as *S*. *glanis* is already breeding in some angling lakes, of which some are unlicensed and not properly managed [15].

Should this occur, the risks to native species, despite not being fully quantified [8, 16] are likely to include disease transmission [8, 13], detrimental impacts of predation on native fish communities [9, 17, 18, 19] on waterfowl [20, 21, 22], and modified food web structure and ecosystem functioning [17, 21, 23, 24]. These potential detrimental impacts suggest that there is a strong requirement for increased education of anglers to prevent further inappropriate introductions and better regulation of extant *S*. *glanis* fisheries to avoid their riverine dispersal [8, 19, 25]. For such schemes to be successful, they need to understand the demographics, motivations and values of the anglers and fishery managers involved [5]. Consequently, this study investigates the socioeconomic characteristics of *S*. *glanis* anglers from a representative selection of their fisheries in England to elucidate human dimensions of non-native species angling and fisheries management [8]. Specific objectives were to: 1) investigate angler perceptions of problems and risks associated with non-native *S*. *glanis* and fishing practises; 2) establish whether there were different motivations among respondents and reasons for any variability in importance of drivers; and 3) explore whether there are any economic benefits and variation in specialist *S*. *glanis* consumerism among respondents.

## Material and methods

Data were obtained from responses to a questionnaire containing 42 questions broken down into five components: 1) social demography; 2) annual angling expenditure; 3) activity trends; 4) motivation; and 5) attitudes and awareness among anglers from a cross section of mixed cyprinid fisheries and the Catfish Conservation Group in the UK. The survey included six fisheries from southern England; Kent, Sussex, Essex, Hertfordshire and two from further north—Yorkshire and Staffordshire. The survey started on 10 May and ended 18 August 2014. A total of 450 questionnaires were distributed by the first author EMAR and by bailiffs and fishery owners using a chain sampling method (snow ball sampling or referral) with the co-operation and written consent of bailiffs and fishery owners who agreed to participate in the study and allow questionnaire data to be published. In addition, an *S*. *glanis* fishery expert from the Catfish Conservation Group distributed and collected questionnaires from anglers and associated members by mail and also by social media posts (Facebook, Twitter). For potential participants, the study relied on good communication from bailiffs and fishery owners to the author EMAR who attended an *S*. *glanis* angling event at one of the fisheries for recruitment, with over 30 participant’s questionnaires collected by the end of the day. Overall, there was a good response rate (90%) from participants fishing at specimen lakes when approached on a one to one basis by EMAR, fishery owners or bailiffs on the day, during random weekend visits. The respondent’s demographics were well representative in average age, marital status, house hold number and home area which matched Office National Statistics (ONS) in the UK and other demographic surveys of recreational anglers [26, 27]. All respondents who participated in the study were over 18 years old, and gave written consent to be involved in the survey and agreed that their responses to be published. A total of 186 respondents completed the survey of which (89%) met the inclusion criteria for the study (n = 166).

The questionnaire was adapted from peer reviewed literature with similar published angler questionnaires [26, 27]. The authors made changes to questions so as to fit the specific research questionnaire which was checked and pretested for content and face validity by several experts in fisheries from the Environment Agency and also Catfish Conservation Group prior to the survey. The questionnaire was approved by the Chair of the Health and Human Sciences Ethics Committee, University of Hertfordshire. For reasons of ethics, cost-effectiveness and statistical reliability of the questionnaire, it was crucial that bias was minimised and random [28], and that responses to the questionnaires were anonymous and confidential. Other limitations were some of the selected fisheries in the study were based in counties surrounding London rather than throughout the UK, which, although reflective of *S*. *glanis* angling popularity in these areas, may cause some regional bias.

The demographic section of the questionnaire was designed to elicit understanding of gender, age, marital status, number in household, angling experience, education, employment and income status of anglers together with possible regional differences. To clarify causal factors related to angler demographic data, respondents were asked to answer from a list of knowledge options to each question, e.g. gender was categorised into male or female, with age ranked into four age groups: 1) 0–29; 2) 30–49; 3) 50–69; and 4) more than 70. Marital status was divided into: 1) single, 2) married or partner; and 3) widowed. Number in the household was categorised into six groups in sequential order. Regions were divided into: Wales, Scotland, East Anglia, South East England, South West England, Midlands and North England. Respondents were asked to select from four options regarding education status; 1) elementary school; 2) technical college; 3) high education college; and 4) university, while employment status was selected from seven categories; 1) unemployed; 2) employed worker; 3) pupil or student; 4) self employed; 5) apprentice; 6) public servant; and 7) retired. Respondents were asked to select from six possible monthly income status groups: 1) < £1000; 2) £1000–1500; 3) £1500–2000; 4) £2000–2500; 5) £2500–3000; and 6) > £3000. The demographic analysis relating to respondents included questions about angling experience of cyprinids and of *S*. *glanis* and they were asked to select from seven options: 1) < 1 yr; 2) 1–5 yr; 3) 5–10 yr; 4) 10–15 yr; 5) 15–20 yr; 6) 20–25 yr; and 7) > 30yr, respectively.

The second section of the questionnaire was designed to obtain data about the annual expenditure on angling among respondents, e.g. annual expenses of equipment and bait, travel, fishing license, membership fee or day ticket. Each question was answered by selection from six possible options: 1) < £100; 2) £100–£300; 3) £300–£500; 4) £500–£1000; 5) £1000–£2000; and 6) > £2000. The number of fishing licenses per angler was also included and respondents could select from the option list the number of licences they purchased annually.

To determine activity patterns and time spent angling, e.g. hours spent actively angling, respondents could select from six options: 1) < 2 hr; 2) 2–4 hr; 3) 4–8 hr; 4) 8–12 hr; 5) 12–24 hr; and 6) > 24 hr. Respondents were asked whether the average time spent fishing from arrival to departure was: 1) < 4 hr; 2) 4–8 hr; 3) 8–12 hr; 4) 12–24 hr; 5) 1–2 days; 6) 2–3 days; and 7) > 3days. In answering the question about days spent fishing in a year, respondents could choose from seven options: 1) < 10 days; 2) 10–30 days; 3) 30–60 days; 4) 60–180 days; 5) 180–240 days; 6) 240–300 days; and 7) > 300 days.

The final section of the questionnaire addressed motivation and awareness among respondents. Questions discerning angler motivation were wide ranging and included: enjoyment of nature, escape, socialising with family or friends, opportunity for adventure and accomplishment, the chance to catch a trophy fish, improve fishing skills and learn new techniques. The challenge and size of catfish were also included as questions in reasons for angling. Respondents selected from a 5-point rating scale with the knowledge options of: 1) not at all important; 2) slightly important; 3) moderately important; 4) very important; and 5) extremely important.

Questions regarding perception and knowledge of risks about *S*. *glanis*, e.g. disease transmission, trophic impact, predation, hybridisation, establishment and dispersal into rivers, non-native fish legislation and information available from angling organisations were included. The respondents were asked for their level of knowledge by answering from a 4-point rating scale of: 1) don’t know; 2) superficially; 3) partially; and 4) completely.

Similarly, questions concerning views about costs of *S*. *glanis* angling, fishing licenses, awareness of adverse impacts from stocking rates, bait and necessity and period of closed season were included. In answering these questions, respondents were asked to select from a list of 4-point rating scale: 1) don’t know; 2) sufficient; 3) too low; and 4) too high.

Descriptive frequency statistics for all categorical independent variables in the questionnaire were determined and the Chi square test (χ^2^) was used to ascertain whether these variables (e.g. gender, age, education status, employment status, income status, angling experience of *S*. *glanis*, hours spent actively fishing, days spent angling in a year, average distance from home to fishery, view of costs in *S*. *glanis* fishing, view of bait allowed in angling and view of period of closed season) were statistically significant. A bivariate analysis was performed to investigate two-way relationships across variables, i.e. regional differences in annual expenditure on bait and equipment, travel, fishing license, membership, day tickets and income status. Bivariate analysis was used to investigate awareness among respondents about risks of disease transmission, trophic impact, predation, hybridisation, establishment and dispersal issues concerning *S*. *glanis* in relation to their knowledge of non-native fisheries legislation and perception of how well informed they were by media and angling organisations. Bivariate analysis was also used to determine respondent’s views of bait permissible in angling and adverse risks of high fish stocking rates in angling lakes relative to individual perception of training and information provided by angling groups and media.

Logistic regression, bivariate analysis and the Chi square test were used to determine the relationships between the binary dependent variable (average time spent pattern (high or low) in angling) and independent motivation variables (importance of fishing motivation to relax in nature and tranquillity, escape from everyday stress and be alone, socialise with family or friends, to experience sense of adventure, excitement or sporting accomplishment, to catch and experience fight of a trophy fish, e.g. *S*. *glanis*, the importance of size or challenge as a catch incentive and to improve fishing skills and learn new techniques). The binary dependent variable, average time spent in angling activity from arrival to departure at fishery was coded as 0 or 1 (0 = average time spent in angling is less than or equal to 12 hr, 1 = average time spent in angling more than 12 hr).

The logistic regression method was calculated using: *ln* (*p*/1-*p*) = *β*_0_+ *β*_*i*_ *X*_i._where *p* = the probability of pattern in time spent in angling; (*p*/1-*p*) = odds of time spent pattern in angling; *β*_0_ = constant; *X*_i_ = vector of independent motivation variables; and *β*_i_ = parameter estimate for the ith independent motivation variable. The individual effects of categorical independent motivation variables on the binary dependent categorical variable were determined, indicating either greater or lesser average time spent angling [29]. All statistical analysis was performed using IBM SPSS statistics version 21, with an α <0.05.

## Results

A total of 450 questionnaires were sent to a cross section of fisheries in the UK that agreed to participate in the study, of which 166 (37%) anglers returned questionnaires with the majority of respondents (70%) from south east England. Social demography among respondents indicated a significantly higher proportion of male anglers (95%, respondents (χ^2^ = 135.54, d.f. = 1, *P* < 0.001). Most *S*. *glanis* anglers were between 30–40 years of age with a higher education college or technical college education (S1 Fig). Typical *S*. *glanis* anglers were employed, earning a moderate income <£30K per annum, with a lower proportion (4%) receiving an income >£36K (χ^2^ = 53.59, d.f. = 25, *P* < 0.001) (Table 1).

**Table 1:** Significance of different socio-economic and demographic characteristics of respondents in the study.

Social demography characteristics	Observed frequency	χ^2^	d.f.	*p-value*
**Gender**:		135.54	1	<0.001
male	158			
female	8			
**Age (yrs)**:		107.74	3	<0.001
<20s	44			
30-40s	87			
50s-60s	19			
60+	2			
**Marital status**:		57.29	2	<0.001
single	44			
married/ partner	71			
widow	4			
**Education:**		30.04	3	<0.001
elementary	44			
technical college	38			
high college	25			
university	6			
**Employment status:**		357.63	6	<0.001
unemployed	6			
employed	102			
pupil/ student	14			
self employed	22			
apprentice	2			
public servant	1			
retired person	6			
**Average annual income (£)**		31.72	5	<0.001
<12,000	10			
12,000–18,000	20			
18,000–24,000	29			
24,000–30,000	14			
30,000–36,000	5			
>36,000	5			
***S*. *glanis* angling experience (yrs)**		269.80	6	<0.001
<1	14			
1–5	91			
5–10	31			
10–15	14			
15–20	3			
20–25	2			
>30	3			
**No of *S*. *glanis* angling trips (per yr)**		47.63	3	<0.001
>3	4			
4–10	57			
11–20	55			
>20	36			
**Average distance from fishery (miles)**		53.27	6	<0.001
<5	9			
6–10	34			
11–20	47			
21–30	32			
31–40	15			
41–50	8			
>50	21			

Variation in angling expenditure among respondents was found across all regions. Annual expenditure on bait and angling equipment were fairly high among total expenses, with bait a main cost; most anglers (54%) in East Anglia spent £300-£500, with fewer respondents (23%) spending >£500–£1000 with similar trends observed in the Midlands and north of England (χ^2^ = 47.58, d.f. = 20, *P* < 0.001). Expenditure on travel, fishing license, membership fee or day tickets annual costs were lower than for angling equipment and bait. Most anglers (50%) in East Anglia spent £100–£300 on travel χ^2^ = 54.15, d.f. = 20, *P* < 0.001) whereas 58% of anglers spent about £100–£300 on membership or day tickets fees (χ^2^ = 42.55, d.f. = 20, *P* = 0.01) (Table 2). The majority of respondents (56%) indicated that the costs for fishing for *S*. *glanis* were acceptable, although (29%) considered outgoings to be too high (Table 3).

**Table 2:** Significant variation in angling expenditure by respondents across different regions in the study.

Angling expenditure (£)	Percentage of respondents (%) from various regions in the UK				χ^2^	d.f.	*p-* value
	East Anglia	South East England	Midlands	North England			
**InAnnual angler income (£)**							
12K–18K	22.2	21.7	50.0	-	36.85	20	0.012
18K–24K	44.4	40.0	-	-			
24K–30K	22.2	15	16.7	-			
30K–36K	11.1	1.7	16.7	66.7			
>36K	-	6.7	-	33.3			
**Annual angling bait and equipment expense (£)**							
>300–500	53.8	37.8	31.3	60	47.58	20	0.000
500- 1K	23.1	25.2	12.5	-			
>1K	11.5	15.3	6.3	-			
**Annual angling travel expense (£)**							
<100	19.2	13.0	33.3	20	54.15	20	0.000
>100–300	50.0	37.0	33.3	-			
>300–500	15.4	35	33.3	-			
**Annual angling license and membership fee/ day ticket expense (£)**							
<100	11.5	8.3	33.3	40.0	42.55	20	0.002
>100–300	57.7	66.1	53.3	20.0			
>300–500	19.2	14.7	13.3	40.0			
500–1000	7.7	7.7	-	-			

Note: d.f.—Degrees of Freedom

**Table 3:** Evaluation of perception and knowledge of significant socio-economic aspects of specialist *S*. *glanis* anglers in the study.

Socio-economic views of specialist anglers	Observed frequency of respondents perception range						
	Do not know	Sufficient	Too low	Too high	χ^2^	d.f.	*p-* value
**In Costs in specialist angling of *S*. *glanis***	18	79	2	41	95.71	3	0.000
**Allowance of unlimited bait in angling**	28	118	5	8	213.25	3	0.000
**Stocking density of fish in specimen lake**	33	100	22	1	140.77	3	0.000
**Adverse risks of high fish stocking density in specimen lake**	83	42	6	13	102.01	3	0.000
**Costs of fishing license**	8	31	10	108	168.83	3	0.000

Note: d.f.- Degrees of freedom

The majority of anglers (43%) spent on average between 12 and 24 hr fishing from arrival to departure, fishing 10–30 days per year, within a 10–30 miles (16–48 km) fishery radius (Table 1). The proportion of time spent angling was significantly related to some aspects in angling motivation, i.e. fishing challenge, to enjoy nature and tranquillity and escape from every day stress and to be alone. Many of the respondents (50%) considered size of *S*. *glanis* highly important ((χ^2^ = 10.42, d.f. = 4, *P* < 0.001), with the challenge in catching *S*. *glanis* an influential factor (χ^2^ = 10.12, d.f. = 4, *P* < 0.001). The need to escape from daily stress and commune with nature were important motivations (*P* = 0.01) (Table 4).

**Table 4:** Logistic regression model in the final model showing significant important fishing motivations affecting average time spent angling among respondents in the study. (Final model included only significant variables at α = 0.05 level).

Motivation variables	Ranking	Estimate	S.E.	Wald	d.f.	*p*-value	Odds ratio
Intercept		1.56	1.52	1.05	1	0.305	4.748
Relax in nature & tranquillity	Very important	4.44	1.80	6.09	1	0.014	84.73
	Moderately important	18.32	13206.86	0.00	1	0.999	90773708.26
	Slightly important	1.26	0.90	1.98	1	0.159	3.53
	Not at all important	1.27	0.68	3.51	1	0.061	3.58
Escape from daily stress & be alone	Very important	-1.86	1.34	1.93	1	0.165	0.16
	Moderately important	-1.45	1.29	1.26	1	0.261	0.24
	Slightly important	-2.42	0.89	7.37	1	0.007	0.09
	Not at all important	-1.04	0.84	1.55	1	0.212	0.35
To catch a trophy fish e.g. *S*.*glanis*	Very important	-35.22	19927.64	0.00	1	0.999	0.00
	Moderately important	1.40	1.18	1.41	1	0.235	4.06
	Slightly important	1.06	0.94	1.26	1	0.261	2.88
	Not at all important	0.74	0.89	0.69	1	0.406	2.10
The challenge in catching *S*.*glanis*	Very important	31.52	19927.64	0.00	1	0.999	4.895E+13
	Moderately important	-2.76	1.31	4.46	1	0.035	0.06
	Slightly important	-2.06	1.09	3.56	1	0.059	0.13
	Not at all important	-0.68	1.04	0.43	1	0.510	0.51
Is size of *S*.*glanis* important?	Very important	-0.14	1.47	0.01	1	0.924	0.87
	Moderately important	-0.42	1.25	0.12	1	0.735	0.66
	Slightly important	-0.29	1.16	0.06	1	0.800	0.75
	Not at all important	1.56	1.52	1.05	1	0.305	4.75

Note: N = 142 selected cases in analysis (missing cases 24, Total = 166). Model chi-square = 44.60, d.f. = 24, *p* = 0.006, -2log-likelihood = 144.04.

d.f.- Degrees of freedom. S.E.—Standard error

Awareness of potential risks and adverse ecological impacts associated with *S*. *glanis* were significantly low, with only partial understanding of impacts and superficial comprehension of non-native fish legislative control measures shown by some anglers (30%) (χ^2^ = 110.98, d.f. = 9, *P* < 0.001). Such poor awareness was possibly linked to inadequate training and information provided by angling organisations and media, as (21%) stated that they were sufficiently informed (χ^2^ = 39.66, d.f. = 9, *P* < 0.001. A high proportion (74%) believed that the use of unlimited bait was good practise, whereas few had reservations about the adverse impacts from bait overloading and fish overstocking in lakes (χ^2^ = 46.89, d.f. = 9, *P* < 0.001). Only few (2%) were adequately informed by angling organisations about the risks from high bait loading (χ^2^ = 33.24, d.f. = 9, *P* < 0.001).

## Discussion

In recent years, there has been an increase in specialist angling for *S*. *glanis* in the UK, with large individual fish imported from mainland Europe and higher frequency in riverine dispersal. To enable the primary ecological risks to be identified and development of more robust measures for non-native species education and management requires understanding of the socio economic factors and levels of ecological understanding among the specialist anglers concerned [30, 31].

The majority of respondents who participated in this study were male, recently specialised in *S*. *glanis* angling (for less than 10 years), aged between 30 and 40 years, and educated to elementary or college level rather than university. The highest proportion earned a moderate income (~ £30K per annum) with limited financial and time resources to spend on angling. Most spent between 10 and 30 days per year angling, living within a 16 to 48-km radius from their preferred fishery. Similar results have been found elsewhere, with anglers specialising in catching large common carp *Cyprinus carpio* in Germany being mostly male [31], fairly young [32, 33] and having less experience than other anglers. They were also prepared to travel longer distances and spend more on angling than older experienced anglers [26]. For these anglers, time spent angling was restricted to approximately 45 to 49 days per year, with a strong influence of constraints imposed by their income and employment. In addition, other studies have indicated that resident anglers (e.g. those living within 20 km of a fishery) will spend a greater number of days angling than non-resident anglers, a factor related to reduced travelling costs [34, 35].

In the present study, there was significant variation in angling expenditure with the greatest expense spent on bait and angling equipment, yet little geographic difference in costs of annual membership or ‘day tickets’ (i.e. the cost to fish during daylight hours) for accessing specimen lakes holding *S*. *glanis*. The mean cost of a day ticket for specimen lakes was approximately £13.50, approximately twice the cost for lakes holding only species of the Cyprinidae family, highlighting the financial attractiveness to a fishery manager by the addition of *S*. *glanis*. As a general rule, many fisheries were run for members, with most having a fixed number of members to restrict angling pressure and preserve angling quality. For these, the mean annual membership fees were approximately £254, although there was variability in, for example, geographic location, intensity of stocking and angling pressure. As recreational fisheries are an integral aspect of rural tourism [1, 36, 37], then increased availability of specimen lakes holding *S*. *glanis* charging higher access fees might contribute increased annual revenues to local economies [38, 39].

It was apparent that the addition of *S*. *glanis* into specimen lakes provided large sized trophy fish for exploitation, good catch rates for anglers and fish hardy to handling stress. This in turn was likely to have resulted in more repeat visits by successful anglers, thus increasing fishery revenue [10]. Indeed, the long-term survival of *S*. *glanis* appeared relatively high in the fisheries surveyed consistent with other studies on *S*. *glanis* [8, 9, 40]. Moreover, their apparent resilience to regular capture and handling contrasted to other freshwater fishes targeted by anglers that appear more vulnerable to angling-related mortality, such as pike *Esox lucius*, zander *Sander lucioperca* and bull trout *Salvelinus confluentus* [35]. Specialist angling for *S*. *glanis* was however, highly seasonal, with catch rates higher in mid-summer than autumn, probably because the species starts foraging at temperatures above 15°C (i.e. in late spring and summer in the UK) [8, 9, 40]. Thus, stocking *S*. *glanis* into UK fisheries appears unlikely to result in increased revenues throughout the year, but instead display highly seasonal returns.

Effective landscape management of recreational fisheries appeared essential for anglers, as increased time spent angling was significantly related to tranquillity, natural surroundings and escape from daily stress, as found elsewhere [5, 12]. In many cases, fish species diversity and abundance were considered a priority by anglers [34], whereas for some, travel costs were the main determinant [35, 41, 42]. Low angling pressure, maintenance of lakes in good ecological status, and sufficient fish abundances to ensure angler satisfaction also tend to be important requirements [12, 34].

In the present study, both fish size and challenge in capturing *S*. *glanis* were also significant factors attracting anglers, as most were ‘trophy’ anglers, thus fishing from a somewhat biased perspective. This becomes more relevant for *S*. *glanis* anglers because catching large fish requires a certain level of competence and experience, because older fish are cautious to bait enticements and capture [43]. This motivation for fish capture as well as the desire to go angling within a naturally attractive setting with tranquillity and solitude, were fundamental needs among the respondents and concur with other studies [26, 32, 34, 35, 44]. Thus, there was a wide range of angler motivations evident from relaxation in idyllic surroundings to competitive targeting of specific fish species for large size and catch challenge, which are crucial in driving introductions of large bodied non-native fish species in the UK, and thus requires more consideration in the development of more robust regulations and policies.

The majority of respondents had little knowledge of the risks posed by non-native *S*. *glanis* stocked into the fisheries, with only partial understanding of the various ecological threats, such as disease transmission, predation, trophic consequences, alteration of community structure, and declines in native species [45]. Similarly, most respondents were unaware of the problems associated with excessive ground-baiting by anglers, which can cause of eutrophication in lakes [12, 46].

Knowledge of non-native fish legislation and fishery management was mostly superficial amongst respondents and related to lack of information. Consequently it seems that *S*. *glanis* dispersal and establishment from a proportion of these lakes into some river catchments is highly probable, irrespective of legislation [14]. In entirety, this suggests that in combination with the lack of extant regulatory and ecological knowledge displayed by anglers allied with their unsustainable angling practises and strong desire to catch large specimen fish within the UK, makes it almost inevitable that *S*. *glanis* will continue to be stocked by financially-minded fishery managers into lake fisheries with subsequent ecological consequences [27, 46]. Consequently, there is an urgent requirement to improve communication and information transfer to anglers from angling organisations, regulatory bodies, policy-makers, researchers and the media about the risks posed by non-native fish species [45] to address these concerns through fisheries management actions [27, 35, 39, 47].

The long-term aim should be to reassess the exploitation and release of large *S*. *glanis* individuals into UK fisheries, which although contributing some socio-economic benefits, we argue there are trade-offs against an increased risk of accelerating their ecological impact following natural dispersal into adjacent river catchments. Prevailing ignorance was detected among the majority of anglers who displayed poor awareness and limited knowledge of any detrimental ecological impacts posed by non-native *S*. *glanis* in angling lakes, non-native fisheries legislation and other unsustainable angling practises, compromised by a lack of information and training available to them from angling organisations. Such a response highlights the need for better dialogue between anglers, angling organisations and fishery management; as present risks related to *S*. *glanis* are underestimated and given the outcomes from this study, non-native fisheries management in the UK requires more stringent regulations. It is highly probable that *S*. *glanis* will increasingly colonize major river catchments, facilitated by warming air and water temperatures predicted for the UK, with their invasion resulting directly from inadequate regulatory control, poor angler education and disreputable fisheries.

## Supporting information

S1 Text Questionnaire used in the study. (XLS)

S2 Text Email correspondence of ethical approval in this study. (DOC)

S1 Fig Significant socio-economic aspects of specialist *S*. *glanis* anglers in the study. (DOC)

S1 Dataset Data obtained from questionnaire in this study. (SAV)
