# Supplementary material for: Socio-economic drivers of specialist anglers targeting the non-native European catfish (Silurus glanis) in the UK
Source: PLoS One. 2017 Jun 12;12(6):e0178805. doi: 10.1371/journal.pone.0178805 (PMC5467846; doi:10.1371/journal.pone.0178805)
Supplement: S2 Text — (DOC) [file pone.0178805.s002.doc]

Hi Ann

I don’t know exactly what PLOS One require – would this email do or do you need a letter from Richard that can be scanned and sent? There are no ethical issues and Dr Southern is happy to approve it.

All the best

Ronni

**From:** Southern, Richard 
**Sent:** 09 December 2015 11:55
**To:** Edmonds-Brown, Veronica
**Subject:** Re: Questionnaire

Dear Ronni

I have reviewed Ann Rees’ questionnaire, and can confirm that from an ethical perspective there is no need for a formal application to the committee as it is essentially fact finding not opinion seeking, and I am happy to take chair’s action and approve the questionnaire for study.

Regards

Dick

--

Dr Richard Southern

Chair, Health and Human Sciences

Ethics Committee (with delegated authority)

University of Hertfordshire

HATFIELD

AL10 9AB

+44(0) 1707286491

M 07900 637112

**From:**"Edmonds-Brown, Veronica" <[v.r.edmonds-brown@herts.ac.uk](mailto:v.r.edmonds-brown@herts.ac.uk)>
**Date:**Friday, 4 December 2015 14:03
**To:**Richard Southern <[r.l.v.southern@herts.ac.uk](mailto:r.l.v.southern@herts.ac.uk)>
**Subject:**Re: Questionnaire

Hi Dick

Am attaching Ann Rees questionnaire. This was carried out when she was working as an independent fisheries consultant before she started her PhD with us. She has just submitted it to PLOS ONE who have asked her for her ethics approval. You said we don’t do ethics approval retrospectively, but that you might be able to provide a letter as Chair of the board of our Ethics Committee to say it’s not breaching any guidelines.

Kind regards

Ronni
