# Supplementary material for: Socio-economic drivers of specialist anglers targeting the non-native European catfish (Silurus glanis) in the UK
Source: PLoS One. 2017 Jun 12;12(6):e0178805. doi: 10.1371/journal.pone.0178805 (PMC5467846; doi:10.1371/journal.pone.0178805)
Supplement: S1 Fig — (DOC) [file pone.0178805.s003.doc]

**Figure 1. Significant socio-economic aspects of specialist *S. glanis* anglers in the study.** (Education n=133, Annual Income n=83, Number of Angling trips n=133 and Average distance from fishery n=166).
